# The Use of Effective Language and Communication in the Management of Obesity: the Challenge for Healthcare Professionals

**DOI:** 10.1007/s13679-021-00441-1

**Published:** 2021-05-18

**Authors:** Sameera Auckburally, Elena Davies, Jennifer Logue

**Affiliations:** 1 Faculty of Health and Medicine, Health Innovation One, Sir John Fisher Drive, Lancaster University, Lancaster, LA1 4AT UK; 2 Department of Paediatrics, Blackpool Victoria Hospital, Whinney Heys Rd, Blackpool, FY3 8NR UK

**Keywords:** Obesity, Language, Communication, Person-first, Paediatric obesity, Healthcare

## Abstract

**Purpose of Review:**

Initial conversations about weight with patients are important to set the tone for future dialogue and management of obesity. There is often reluctance in raising the topic of overweight or obesity in consultations. We aimed to evaluate literature to discover the perceived barriers to optimal discussion about weight status and preferred weight-based terminology for adults, adolescents and parents of younger children.

**Recent Findings:**

Fear of offending patients, insufficient training and lack of knowledge of referral pathways were identified as factors hindering healthcare professionals’ ability to discuss weight with patients. Neutral terms, such as ‘weight’, were preferred by patients, with ‘fat’ and ‘obese’ viewed as undesirable and stigmatising words.

**Summary:**

There is a need for greater support and provision of specific training, including education on communicating weight status, for those involved in the management of obesity. More research is necessary to assess the impact of interventions to improve initial discussions with patients about weight.

## Introduction

It has been established that people with obesity face prejudice and discrimination as a consequence of their weight [1, 2]. This is pervasive across age groups and different settings, including schools, workplaces, the media and in healthcare [1], with stigmatising experiences having negative consequences on psychological well-being and physical health [3].

It has been reported that people with overweight and obesity have differing experiences of healthcare compared to those with a lower body mass index (BMI) [4], with patients with obesity citing previous disrespectful treatment and negative attitudes from healthcare professionals as barriers to seeking further medical care [5]. Stigmatising communication in the medical setting can exacerbate the problem. The language healthcare professionals use in weight-based consultations can have an impact on how one feels about their condition [6]. Increasing evidence of weight bias in healthcare has led to policy recommendations and consensus statements outlining the importance of respectful communication about weight with the use of non-stigmatising language [7–9].

This review article aims to evaluate the literature on the perspectives of patients and healthcare professionals on the topic of weight-based communication in clinical practice, both in adult medicine and in the paediatric setting. Interventions to reduce stigma and improve communication of weight status to patients will also be discussed in this review, along with future research needs.

## The Views of Patients

Stigma surrounding obesity and weight results in challenges for both healthcare professionals and patients when it comes to raising this topic in a consultation. Qualitative data from patient interviews indicated the language used by health professionals is deemed crucial by patients, as it influences their consultation experience and how comfortable they feel discussing their weight [10].

By way of questionnaires, studies conducted across the UK, the USA and Australia have investigated the desirability of various terms that may be used by clinicians when discussing weight issues with their patients [10–13, 14•]. The terms ‘weight’ and ‘BMI’ were found to be the preferred and least offensive terms, whilst ‘fatness’ was reported to be the least desirable word of those offered [11, 12]. Other terms such as ‘obese, ‘large size’ and ‘excess fat’ were also rated as undesirable [10–13], with ‘morbidly obese’, ‘fat’ and ‘obese’ viewed as the most stigmatising and blameful words [14•]. Indeed, qualitative studies found many patients rejected the label ‘obese’ due to feelings of stigma and discrimination [15, 16]. Preferred and disliked weight-based terminology can be found in Table 1.

**Table 1:** Language preferred by patient groups in weight-based consultations

	Preferred language	Disliked language
Adults [10–13, 14•]	Weight BMI	Fatness Obese Large size Fat Excess fat
Adolescents [17•, 18•]	Weight Weight problem Plus-size	Obese Large Fat
Parents of younger children [18•, 19–21]	Gaining too much weight Too much weight for his/her health	Overweight Obese Fat

Weight-based terminology perceived as more desirable from patients’ perspectives is not always used by health professionals. One patient, interviewed as part of a study conducted in rural areas of Australia, described a personal experience in which the language used by their doctor bought them to tears [22]. A negative experience such as this may lead to a patient avoiding the topic of obesity in future consultations and therefore missing the opportunity to be referred to support services. This stresses the importance of outlining the most desirable language based on patients’ perspectives.

A healthcare professional’s choice of language and communication with their patient is likely to be influenced by their attitudes and personal beliefs. Malterud et al. used focus groups to explore patients’ personal experiences with weight management in general practice [23]. Patients often felt a negative attitude towards obesity was held by the health professionals caring for them, with feelings of guilt, shame and humiliation described by patients believing they were being blamed for their weight issues by their clinician [23]. Weight bias in healthcare can result in the avoidance of patients engaging in conversations regarding obesity.

Several studies have conducted qualitative interviews with patients on their experiences of the management of obesity in primary care [23–25]. Patients reported a need for health professionals to receive further training in the management of patients with obesity [23–25]; there was an overall lack of knowledge of support services [23]. In addition, patients complained of insufficient engagement from clinicians in the treatment of obesity. These factors led to patients being disappointed by the lack of enthusiasm, attention and support offered by their general practitioner, and some patients attributed this to their poor success in being able to make and sustain lifestyle changes [23]. Patients also stated that they had received contradictory advice from healthcare professionals on the management of obesity, which created a great deal of frustration and decreased their motivation to manage their weight [25].

Some patients appeared motivated in seeking help from their clinician for weight-related issues, however faced barriers during the process [22, 24, 25]. Patients discussed a reluctance amongst general practitioners to enter conversations regarding obesity [25], whilst other patients wishing to raise weight-related concerns identified restrictions on consultation time as a barrier in doing so [22]. Many patients described feeling dismissed and rushed by their clinician [22]. The lack of regular consultation appointments was also expressed as an important issue for those who wanted an individual plan to guide their weight loss [24]. This research indicates barriers to weight discussion still exist even for patients who raise concerns themselves.

## The Views of Healthcare Professionals

Interviews and focus groups investigating the challenges accompanying the management of obesity from the perspective of healthcare professionals found similar barriers to those reported by patients [25–32]. One is a belief that the interventions available for the management of obesity are not effective [26–28, 32]. Some professionals attributed this to other medical conditions that their patients may have. For example, a patient with limited mobility or one that takes medications associated with weight gain may find it challenging to reduce their weight [27]. Other professionals held a bias towards patients with overweight, believing they lack the motivation and willpower to succeed in a weight management programme [25–27, 32].

Interestingly, qualitative research gathered from medical professionals across the UK, the USA, Sweden and the Netherlands demonstrated a shared opinion that obesity is not a disease and therefore not within a clinician’s responsibility to address or manage, unless there was an associated co-morbidity [25, 27, 28, 30]. Ideas regarding the prevention of obesity at a public health level were mentioned; however, the management for current obesity was seen as less important [27].

Health professionals across the UK, the USA and Sweden perceived lack of skills, knowledge and guidance as barriers in discussing obesity with their patients [25, 27, 28, 30, 32]. Clinicians mentioned they were fearful to raise the issue of obesity as it may offend patients or have detrimental effects on their relationship [25, 29•, 31, 32]. In addition to a lack of skills required to hold a conversation about obesity, professionals also admitted to lacking knowledge of the referral options for patients with obesity [32]. Due to the recognition of gaps in their knowledge and skills, many professionals expressed low confidence in their capabilities and a desire to gain better training and support in this area [27, 29•, 31].

Furthermore, results from research, including general practitioners, nurses, physiotherapists, dieticians and community nurses working in the Netherlands, indicated health professionals may be avoiding conversations regarding obesity if they believe their patients are not themselves aware of their weight problems [25]. A physiotherapist described multiple consultations in which patients were surprised to discover their BMI was categorised as unhealthy [25]. Concerns about initiating an unexpected conversation on a sensitive topic also add to healthcare professionals’ fears of offending their patients [25].

A good doctor-patient relationship was viewed as important by patients and was also valued by doctors as an aspect that provides them with the confidence to engage in conversation with patients about obesity [31]. General practitioners reported having knowledge of a patient’s social circumstances allows them to determine if a patient is ‘ready’ to discuss weight issues and how successful they may be on a management programme [31]. However, not all clinicians have the advantage of being familiar with their patient and having knowledge of their social situation. This is therefore another possible barrier to initiating a discussion about obesity that needs to be addressed.

## Interventions

As outlined previously, both patients and healthcare professionals have various concerns surrounding communication on the topic of obesity in the healthcare setting. The issues described may lead to neglect in discussions about obesity and could prevent patients receiving the support and management they need. This highlights a requirement for these concerns to be recognised and challenged, so clinicians can be confident communicating with their patients in a way that is empathetic and sensitive from the viewpoint of patients.

Interventions to reduce these issues have been implemented in clinical practice, with many focusing on educating health professionals to reduce the stigma surrounding obesity [33••, 34]. O’Brien et al. conducted a study educating students enrolled in a health promotion/public health undergraduate degree programme on how uncontrollable factors, such as genetics and socioeconomic conditions, are associated with obesity [33••]. Its results found a reduction in anti-fat prejudice within the education group compared to the control group [33••]. The same study also showed that providing education focusing on the more controllable causes of obesity, such as diet and exercise, can lead to an increase in anti-fat prejudice [33••]. This demonstrates that both stigma and prejudice can be influenced by education, and the importance of including uncontrollable causes of obesity within education must not be overlooked.

Research by Kushner et al. also showed an educational intervention to likely be beneficial for the management of obesity in clinical practice [34]. After education, study participants showed an increase in their empathy towards obesity and a reduction in obesity stereotyping [34]. The largest improvement was seen in the participants’ confidence levels during interaction with a simulated patient, with improvements in empathy and confidence still apparent one year after the educational intervention [34]. Improving factors such as these is likely to lead to improved communication between patients and clinicians and a higher level of self-efficiency amongst health professionals to refer patients to weight support services, further emphasising the value of educational interventions for current and future health professionals.

Training programmes have also been used to address the issues that stem from a lack of skills and knowledge regarding obesity amongst health professionals [35, 36••]. A programme, which provided interactive training courses, to twenty-nine family practitioners on monitoring and treating obesity, found an association between training and the referral of patients to support services [35]. In contrast, Jay et al. conducted a study to assess the impact of the *5 As counselling* framework, an evidence-based training programme on counselling patients with obesity [36••]. However, this did not find professionals who had received training to be more likely to counsel patients with obesity than those who had not received training [36••]. Interestingly, regardless of the self-efficacy of clinicians to counsel or refer patients, both studies found that an increase in the quality of counselling for patients was evident after completion of the training course [35, 36••]. The differences found between these studies highlight the need for further investigation into the most effective training techniques. Hopefully, future research will clarify the techniques that are best able to improve the communication and management of patients with obesity. By implementing these techniques in clinical practice, the satisfaction of both patients and health professionals in the communication surrounding obesity can be increased.

## The Views of Paediatric Patients and Families

Weight-based victimisation in the paediatric population tends to stem from peers, families and teachers. These stigmatising experiences in their formative years can have long-term consequences on physical and mental health, namely maladaptive eating behaviours, weight gain, low self-esteem and depressive symptoms [2]. Weight labelling can initiate the processes of stigma; hence, it is important that healthcare professionals appreciate the long-lasting effect of words and use non-biased language [37].

Puhl et al. found that American adolescents prefer healthcare providers to use ‘weight’, ‘weight problem’ or ‘plus size’ when discussing body weight. ‘Obese’, ‘large’ and ‘fat’ received lower preference ratings [17•]. Whilst ‘curvy’ was a term preferred by females if used by their family members [38], it received the third lowest preference rating if used by healthcare professionals [17•]. Interestingly, no words achieved ratings greater than 4 on the 5-point rating scale and most words received a mean score in the region of the mid-point of the scale. This could be in part due to the sample of participants having high internalised weight stigma scores and potentially increased sensitivity to even more neutral weight-based terms. With varying preferences noted according to gender, BMI and extent of internalised weight stigma, it may be prudent for clinicians to acknowledge this and ask adolescents about their language preferences [17•, 39•].

There is a paucity of research into younger children’s perspectives on weight-based terminology, with two studies in the Netherlands identified [18•, 40]. 44.6% of children had experienced a clinician using a term that they disliked [18•]. Many words, such as ‘adiposity’, ‘BMI’ and ‘morbid obesity’, were unfamiliar to the majority of children. ‘Fat’ was disliked the most by children [18•]; its appropriateness was doubted in the healthcare setting, with many reporting negative connotations from previous bullying [40].

All parents that had a child with overweight reported that the choice of words mattered, with the term ‘gaining too much weight’ preferred over ‘overweight’, ‘fat’ and ‘obese’ [19]. This has been echoed by other research finding the terms ‘fat’, ‘obese’ and ‘chubby’ to be viewed as the most stigmatising and blaming by parents [20]. There was agreement amongst English- and Spanish-speaking Latino parents in the USA that the term ‘too much weight for his/her health’ (‘demasiado peso para su salud’) was the most motivating, but also acknowledged that any weight-related word could potentially be viewed as insulting, rather it is *how* it is said that can determine how offensive it is believed to be [21]. If parents believed their child to be stigmatised about their weight by a doctor, 37% would feel upset and embarrassed and 24% would avoid future appointments, highlighting the importance of sensitivity in the consultation [20].

When addressing the topic of overweight or obesity in a child, some parents strongly believe it is inappropriate for a clinician to do so when meeting a family for the first time [39•]; 68% of parents would prefer clinicians to outline their concerns with the child present, with the hope it would convey the importance of the problem, albeit using a sensitive approach. Of those that disagreed, 17% expressed concerns about lowering the child’s self-esteem and increasing the risk of eating disorders [19].

Open-ended questions and a respectful tone were highly endorsed by children and parents when discussing weight [39]. Female adolescents with overweight or obesity reported being more receptive to clinicians that included them in the conversation and expressed interest in their lives [41••]. The same study noted that participants were highly sensitive to weight-related communication, which often left them feeling insecure and upset. They much preferred a gentle, encouraging approach with a changed focus from weight management to health management [41••].

## The Views and Experiences of Healthcare Professionals in the Paediatric Setting

With the rising prevalence of paediatric obesity, healthcare professionals are increasingly discussing weight-based health with young patients and their families. These consultations can be perceived as a challenge, with some general practitioners reluctant to have the ‘difficult conversation’ with parents and children [42, 43]. 86.7% of school nurses in Missouri also believed counselling families about weight loss was difficult, but not inconvenient (51.9%) [44].

Johnson et al. found that school nurses in Solihull, England, felt introducing the subject of obesity as particularly problematic, either in person or via written correspondence [42]. In England, letters are sent to parents after a national measurement programme is conducted in primary schools. These letters detail the child’s body mass index and related health risks [45]. School nurses had faced backlash from some parents upon the receipt of these letters, which then acted as a barrier to further meaningful weight-related discussions and referral to lifestyle interventions [42].

Other healthcare professionals have expressed a fear of parental reaction as limiting their capacity to counsel on weight as they were often worried that parents would feel offended and personally judged on their lifestyle or parenting skills [46, 47]. This would lead to avoidance of the topic entirely by a few healthcare professionals to prevent jeopardising the professional-parent relationship [48].

General practitioners acknowledged a role in raising the issue of a child’s weight but felt time constraints in a busy surgery often prevented them from doing so [43]. Time was highlighted as a common barrier to meaningful discussions about weight amongst other healthcare professionals too [49–50, 51••]. This was supported by research showing that paediatricians that spent more time with families during routine well-care appointments were more likely to counsel on the maintenance of a healthy weight [52].

Limited experience and lack of training have also been highlighted as barriers to addressing childhood obesity [53]. School nurses described a perceived lack of knowledge in leading a motivational conversation about weight management with families [54]. General practitioners in Australia strongly felt that they should offer treatment to children with overweight or obesity rather than referring to others, but only 29% felt professionally well prepared to manage childhood obesity [55] and half of the clinicians in rural US family practices reported that they did not have enough expertise to create a structured dietary plan [56]. Healthcare professionals generally expressed concern about the lack of access to clear guidance or referral pathways leading to inconsistent practice based on individual judgement [32, 53, 57].

In terms of weight-based language preferences, a Dutch study reported that healthcare professionals used the terms ‘overweight’, ‘BMI’ and ‘healthy weight’ most often in consultations with children and parents [18•]. Interestingly, healthcare professionals gave the highest preference rating to ‘BMI’, a term that the majority of children were unfamiliar with. This was the only study found that identified healthcare professionals’ preferences of weight-based terminology in paediatric clinical practice; interestingly, it noted the differences in preferences between children, parents and clinicians, warranting further research in this area.

Healthcare professionals regarded the use of objective tools, such as growth charts, as important in presenting the issue of weight in a less judgemental fashion [48, 58••, 59]. Another factor viewed as a facilitator in initiating the discussion of weight was having an established strong relationship with parents [60]. General practitioners noted that the conversation about weight with families should be done ‘carefully and subtly’, an approach that had helped had been to move the discussion from presenting symptoms, such as joint pain, towards how weight may affect those symptoms and how losing weight could potentially improve them [42].

## Interventions and Recommendations within the Paediatric Setting

Conversations with young people about weight have the potential to impact their physical and mental health for the rest of their lives. It is therefore crucial that healthcare professionals ensure these discussions are handled appropriately.

Interviews with healthcare professionals [46], cross-sectional surveys of parents [19] and focus groups with young people and their caregivers [39•] have supported involving both children and parents in consultations regarding weight. This should ideally be with a healthcare professional that has a longstanding relationship with the family in order to engage all relevant stakeholders in affecting change in the household [61, 62]. However, all clinicians, regardless of the duration of relationship with the family, have a responsibility to initiate weight-related discussions as early as possible in order to prevent obesity [63].

In order to mitigate weight stigmatisation, in 2017, *The American Academy of Pediatrics* made recommendations for paediatricians, which included the importance of recognising the complex aetiology of obesity to help prevent stereotyping individuals with overweight or obesity [8]. They also advocated for the use of person-first language and neutral words when describing weight. It may be appropriate to consider asking adolescents and parents on their preferred terms when discussing weight, due to preference variations amongst different cultures and genders [17•, 21, 38]. Objective communication tools, such as growth charts, may help in presenting the child’s weight status in a visual, non-judgemental manner to aid discussion [58••, 59, 64].

Greater support and provision of specific training, including education on communicating weight status, for those involved in the management of paediatric obesity would be incredibly useful, given that limited knowledge and lack of training were widely perceived as barriers to effective communication [53, 57, 65]. Training concentrating on developing confidence in having conversations about weight would allow healthcare professionals to become more proficient in raising the issue with children and their families.

There has been a lack of research into the impact of interventions to improve initial discussions with families about weight. There have been more studies, however, assessing motivational interviewing in paediatric obesity, which is a patient-centred communication style used to modify health behaviour [66, 67]; it has been found to improve BMI *z*-scores [68, 69] and increase autonomous motivation in engaging in a healthier lifestyle [70]. Due to its use as more of a treatment with ongoing clinician follow-ups, it is outside the remit of this review, however improving training in the motivational interviewing technique could improve the confidence in healthcare professionals’ ability to discuss weight, therefore increasing the ease of bringing up the topic opportunistically.

## Conclusion

In both paediatric and adult obesity management, there were similar concerns raised from patients and healthcare professionals surrounding consultations about weight. The lack of training and knowledge of referral pathways hindered clinicians’ ability to bring up the topic of weight. Fear of offending patients and families added to the perceived challenge of discussing weight with patients.

It is important for healthcare professionals to seek permission to discuss weight. Patients advocated for a sensitive and respectful approach, with the use of neutral terminology, such as ‘weight’, rather than ‘obese’ or ‘fat’. It may be sensible to ask patients about their language preferences, especially as varying preferences were noted amongst adolescents of different genders and according to the extent in which they internalised weight stigma.

Educating healthcare professionals on the underlying causes of obesity, including the many uncontrollable factors, can help to reduce the prejudice towards patients with obesity and increase confidence in discussing weight with them. Training directed at developing skills, knowledge and confidence in having conversations about weight would allow for healthcare professionals to more easily raise the topic with patients and families.
